# Large language model exposure and precarious occupations: Unpacking relationships in the Canadian labor force

**DOI:** 10.5271/sjweh.4312

**Published:** 2026-09-01

**Authors:** Arif Jetha, Qing Liao, Peter Smith, Viet Vu, Aviroop Biswas, Brendan Smith, Faraz Vahid Shahidi

**Affiliations:** 1 Institute for Work & Health, Toronto, ON, Canada.; 2 Dalla Lana School of Public Health, University of Toronto, Toronto, ON, Canada.; 3 The Dais, Toronto, ON, Canada.; 4 Public Health Ontario, Toronto, ON, Canada.

**Keywords:** AI exposure, job quality, labor market inequity, large language model, precarious work, workplace

## Abstract

**Objective:**

The adoption of digital technologies has historically impacted the most precarious occupations and contributed to widening labor market inequities. Large language models (LLM) may reshape this relationship. This study examines the association between occupational exposure to LLM and occupational precarity.

**Methods:**

Using Canada’s Labour Force Survey, occupational exposure to LLM and four dimensions of precarity (contractual instability, earnings inadequacy, schedule unpredictability, working-time mismatch) were examined. A multidimensional index was developed to summarize an occupation’s overall exposure to precarity. Four multivariate linear regression models with cluster-robust standard errors estimated the associations between LLM exposure and each dimension of precarity. A fifth multivariate model examined the relationship between LLM exposure and the multidimensional precarity index. Utilizing model coefficients, mean estimates of occupational LLM exposure were produced.

**Results:**

Using the multidimensional precarity index, our analysis showed that occupations characterized by low exposure to precarity had a significantly higher mean LLM exposure [mean 0.386, 95% confidence interval (CI) 0.356–0.417] compared to occupations with medium (mean 0.258, 95% CI 0.221–0.295), high (mean 0.260, 95% CI 0.194–0.328) or very high precarity (mean 0.205, 95% CI 0.136–0.275). Apart from earning adequacy, LLM exposure was also lower among occupations using each separate dimension of precarity.

**Conclusion:**

Occupations most likely to be exposed to LLM are those where precariousness is lowest. These occupations have previously been sheltered from technological change. There is a need of examine the impacts of LLM on workers in job where the technology is prominent.

The precariousness of an occupation is a critical determinant to the health and safety of workers (1–3). Precarious occupations reflect the accumulation of multiple poor employment conditions including job instability (eg, temporary employment), earnings inadequacy (eg, low wages), schedule unpredictability (eg, irregular hours), working-time mismatch (eg, involuntary part-time employment) or a lack of regulatory protections (eg, no union representation) (3, 4). Studies conducted in high income countries document that a growing number of workers are employed in occupations which are characterized as precarious (5–8). Understanding the factors associated with precarious occupations and taking steps to improve employment conditions can have considerable health and economic implications for workers. When compared to those working in occupations with standard work arrangements (ie, full-time and permanent jobs), workers in precarious occupations are more likely to face hazardous working conditions and greater injury risk (2, 9). A recent analysis of 231 307 lost-time compensation claims in Ontario, Canada, over a four-year period, found that greater exposure to temporary employment, low wages, irregular work hours or involuntary part-time work was associated with a greater workplace injury risk. The study also found that occupations with a high level of exposure to ≥2 dimensions of precarity were associated with a nearly threefold risk of injury or illness when compared to workers in occupations facing less (3) precarity. Also, research highlights that financial insecurity and economic unpredictability faced by workers in precarious occupations are associated with greater stress and anxiety and working conditions, which are harmful to worker physical and mental health (3, 10–12). Indirectly, being employed in a precarious occupation can interrupt access to safe housing, nutritious food and other resources known to shape health (2).

Past examples show a relationship between digital technology use at work and precarious occupations (5, 13). The automation of work was most prominent among occupations with repetitive job tasks and low skill requirements, which have been amongst the most precarious (6). Yet, evidence suggests a nuanced pattern; automation technologies can improve working conditions for some workers while simultaneously deepening precarity for others (14). As an example, a study from Japan found that the use of robots to perform job tasks within the nursing home sector alleviated demands on full-time nursing staff, but simultaneously increased the employer’s preference for flexible, non-regular and part-time nurses (15). The increasing motivation for firms to turn to AI systems to perform job tasks in different occupations has created a need to examine how the technology may reshape employment conditions and broader economic arrangements tied to worker’s access to equitable, fair and safe employment. Recent labor market data show that, in most cases, the adoption of AI systems have not contributed to a significant displacement of workers (16). Instead, AI systems may be currently used to complement workers.

In this study, we focus specifically on LLM, a specific form of AI, which utilize machine learning techniques, especially deep learning and neural networks, to recognize patterns, context, and meaning in language. LLM are increasingly being introduced in workplaces to perform job tasks such as language and information synthesis, documentation, communication, and rule-based cognitive problem-solving that are most often clustered in occupations with higher quality employment conditions. LLM can be used to summarize and synthesize vast amounts of information (eg, legislative analysis tools), manage customer inquiries (eg, chatbot customer service applications), support organizational decision-making (eg, resume screening and job matching platforms) and generate technical and creative content (eg, content ideation tools) (17–21). LLM may currently be less likely to be used to perform job tasks requiring intensive interpersonal or physically unpredictable skills (20). LLM may represent a valuable tool to partially automate job tasks performed by workers in knowledge-intensive and highly-skilled occupations (20). There is a small body of research which signal an inverse relationship between occupations that are precarious as well as exposure to LLM (ie, where LLM can be used to perform tasks that make up an occupation). Eloundou and colleagues (18) recently found that just under 20% of occupations in the US consisted of job tasks in which 50% are exposed to LLM. Occupational exposure to LLM was highest among occupations that were characterized by higher wages (one indicator of whether an occupation is precarious or not) (18). Similarly, a study of LLM exposure in Latin American countries found that occupational LLM exposure was highest among workers with greater educational attainment and income (22). Indeed, these previous studies underscore the need to study the relationship between the impact of LLM on occupations through a multidimensional lens of precarious occupations. An analysis of Canadian workers using data from 2022–2025 in which there was a rapid proliferation of generative AI tools, showed that occupations with high exposure to AI and where AI would be most complementary to workers were also more likely to be characterized by full-time work hours and permanent work arrangements (23).

Using data from Canada’s national Labour Force Survey (LFS) and through the analysis of occupational characteristics, our study aims to build an evidence base on the relationship between the extent to which an occupation is characterized by precarity and its exposure LLM. Our study answers the overarching research question: Are components of precarious occupations and overall precarity more or less likely to be exposed to LLM when compared to occupations that are characterized by less precarity? By answering this question, our research deepens an understanding how emerging AI systems impact the quality of work and patterns of labor market inequity.

## Methods

### Data

We conducted a pooled cross-sectional analysis of four years of Statistics Canada’s LFS (2021–2024). The LFS is a nationally-representative cross-sectional monthly survey of Canada’s household population (15 years of age) with an approximate sample of 100 000 workers built using probability sampling (24). Excluded from our analysis were Canadian Territories, which utilize a separate survey methodology. We also excluded individuals who were not employed, self- employed, living on reserves and other Indigenous settlements, full-time members of the Canadian Armed Forces, and living in institutions and households in extremely remote areas with very low population density at the time they answered the survey.

Responding to the survey is mandatory under the Statistics Act in Canada, and data is collected directly from study participants. Statistics Canada manages missing data prior to the dataset being accessed by individual research teams utilizing several imputation approaches including carry-forward, deterministic and donor imputation (24).

The LFS uses the National Occupational Classification (NOC; 2021), a standardized system to classify Canadian occupations based on the type of work performed, including main duties, educational requirements, and other information (25). Using sample weights produced by Statistics Canada (24, 26), weighted monthly LFS cycles were combined to produce annual estimates. Our analysis was conducted at occupational level and not on specific workers.

The data is made available to researchers in compliance with the Statistics Act (1985), ensuring the protection of all personal and confidential information (27). Ethics approval from an institutional research ethics board was not required, as this study involved secondary analysis of data provided by Statistics Canada and satisfied the conditions of Article 5.5A of Canada’s Tri-Council Policy Statement 2 on the Ethical Conduct of Research Involving Human Subjects (28).

### Independent variables: precarious occupations

Using an approach developed by Shahidi et al (3), we examined four dimensions of precarity using items collected in the LFS: contractual instability (ie, temporary employment), earnings inadequacy (ie, low wages), schedule unpredictability (ie, irregular hours) and working-time mismatch (ie, involuntary part-time employment). Temporary employment was assessed using the question: ‘Is your job permanent?’. Exposure to low wages was assessed using the question: ‘What is your hourly rate of pay?’ and we used Statistics Canada’s definition of low wages as earning less than two-thirds of the median hourly wage (29). Exposure to irregular hours was assessed using the question: ‘Does the number of paid hours you work vary from week to week?’. Exposure to involuntary part-time employment was assessed using the pair of questions: ‘How many paid hours do you usually work per week?’ and ‘Did you want to work 30 or more hours per week?’. Involuntary part-time work was classified as working <30 hours/week and wanting to work ≥30 hours /week.

Temporary employment, low wages, irregular hours and involuntary part-time employment were first coded as binary variables determining whether a dimension was present or not. The proportion of workers exposed to a given employment condition was then calculated within each of the four-digit occupational titles. All occupations were rank ordered according to the proportion of workers exposed to a given precarious occupation dimension. Occupations were then grouped into four quartiles of exposure with the first quartile (Q1) representing the occupations with the lowest probabilities of exposure and the fourth quartile (Q4) representing the occupations with the highest probabilities of exposure. Given the number of workers in occupations in the first and second quartiles of exposure to involuntary part-time work were small, these two levels were combined in the final stage of model development. Finally, a multidimensional precarity index was developed to provide an overall summary of an occupation’s exposure to precarity, assessed in terms of the number of times a given occupation was assigned the highest possible exposure level (ie, precarity item assigned to Q4) across the four dimensions. We distinguished between occupations with ‘low’ (ie, never assigned to Q4), ‘moderate’ (ie, assigned to Q4 once), ‘high’ (ie, assigned to Q4 twice) and ‘very high’ overall precarity exposure (ie, assigned to Q4 three or four times).

### Dependent variable: Occupational LLM Exposure

We used the novel LLM exposure measure by Eloundou et al (18) to examine the impact of the technology on occupations. The measure was developed using the US O*NET 27.2, which is an American occupational information database describing nearly 1000 occupations and occupation-specific information via standardized job-oriented descriptors and worker-oriented descriptors (30). O*NET describes 19 265 core or supplemental job tasks (where supplemental tasks were weighted half as much), associated with 2087 detailed work activities (DWA) across 923 standardized occupations (where a DWA may appear in multiple occupations).

Eloundou et al define LLM as software that is applied to text, code, and images. Their definition and analytical approach are not tied to a particular model or model provider but refers to a broad suite of models that can be accessed through a chatbot or application programming interfaces. Using this definition, they created a rubric to assess occupational LLM exposure based on the share of an occupation’s job tasks where access to an LLM alone or with a simple interface would lead to 50%-time savings (based specifically on manual human coding) and the share of an occupation’s tasks where additional software is needed on top of an LLM to realize the 50%-time savings. The rubric was applied to each DWA and job task by a team of human labelers. Results were subsequently aggregated at the occupational level to produce an overall score representing the proportion of job tasks within an occupation that can achieve significant time savings by utilizing an LLM. Although the measurement tool was designed in the US, it’s characterization of occupations according to the extent to which they can be affected by AI has been applied to labor market data in different countries.

To estimate LLM exposure at Canadian occupation-level, we cross-walked the 8-digit O*NET codes to 5-digit Canadian NOC Codes. The concordance between the standardized occupation codes used in the O*NET database and the NOC system used in the LFS was facilitated through Codage Assisté des Professions et Secteurs d’activité – Canada, which provided an actual concordance across coding systems for 350 of 500 NOC codes (one-to-many) (31), and Statistics Canada, which provided a theoretical crosswalk for the remaining 162 NOC codes (one-to-one) (32). We then calculated an occupational-level score for each of the 512 occupations (two occupation codes of Canadian Armed forces were excluded). The occupational-level score represents a continuous outcome variable reflecting the share of tasks within an occupation exposed to an LLM. Higher scores indicate more exposure to LLM within an occupation. The theoretical total range of the score is 0–1 with a score of 0 indicating no tasks within the occupation that could be performed by LLM and 1 indicating that all tasks within the occupation can achieve significant time-saving by utilizing LLM, though no occupation in our analysis reached this maximum.

### Covariates

For our analysis, we examined the following occupational characteristics in the LFS, including gender (proportion of women workers in an occupation); age group (proportion of workers aged 15–24, 25–34, 35–44 years, 45–54, ≥55 years in an occupation); educational attainment (proportion of workers with high school or less/some post-secondary, trades certificate or diploma, college or bachelor’s degree, or above bachelor’s degree); industry categorization using the North American Industry Classification System (NAICS) [goods-producing (11–33) or service-producing industry (41–91)]; and provincial composition.

### Analysis

To answer our research question, all analyses was carried out at the occupational level. Descriptive analyses estimated the mean proportion of each covariate across occupations and estimated the mean LLM exposure score across each quartile of the four precarious occupation dimensions and the multidimensional precarity index. Additionally, we computed correlations between the proportion-based covariates (eg, percentage of women workers in an occupation) and the occupational LLM exposure score. Univariate models were then conducted to examine mean differences in occupational LLM exposure across each quartile of the four precarious occupation dimensions and the multidimensional precarity index. Four multivariate linear regression models were estimated to assess the associations between LLM exposure and each precarious occupation dimension, adjusting for study covariates. Finally, a fifth multivariate linear regression model was estimated to examine mean differences in LLM exposure across each level of the multidimensional precarity index indicator. Several model assumptions were tested. The Breusch–Pagan test indicated the presence of heteroscedasticity across the set of regression models (P<0.001–0.03). To address non-constant error variance, cluster-robust standard errors were employed for univariate and multivariate linear regression models. Residual diagnostics were conducted to assess model adequacy. Normality of residuals was assessed using Q–Q plots and the Shapiro–Wilk test. There was some evidence of models showing statistically significant departures from normality; visual inspection suggested that these deviations were minor. Given the large number of clusters (512 occupations) and the use of cluster-robust standard errors, valid inference does not rely on residual normality. Linearity was evaluated by plotting residuals against each continuous predictor and the fitted values; the lack of apparent nonlinear patterns and the approximately symmetric distribution of residuals around zero suggested that the linearity assumption was satisfied. Potential outliers and influential observations were examined using leverage values and Cook’s distance. Although several observations were flagged for further inspection, manual review did not identify any extreme or unduly influential values, and no observations were excluded from the analyses.

Utilizing coefficients from each multivariate model, mean estimates of occupational LLM exposure were produced with corresponding 95% confidence intervals (CI) across levels of each of the respective precarious occupation dimension and across levels of the multidimensional precarity index. To assess the robustness of the quartile-based classification of precarious occupations, a sensitivity analysis was conducted by reclassifying each precarious occupation variable using tertiles. The multidimensional precarity index was redefined according to the number of indicators at the highest tertile level. Cluster-robust standard error regression models of the multidimensional precarity index on LLM exposure scores, controlling for covariates, was estimated. All analyses were conducted using SAS version 9.4 (SAS Institute, Cary, NC, USA).

## Results

The mean occupational LLM exposure value was 0.34 (minimum=0; maximum=0.84) across the four LFS survey years. A breakdown of LLM exposure values for each Canadian occupations is available in the supplementary material, XXX (supplement 1). Analyses show a negative relationship between occupational precarity and LLM exposure. Table 1 presents unadjusted means of occupational LLM exposure scores across each dimension of precarious occupations (ie, temporary employment, low wage, irregular work hours and involuntary part-time work). Table 1 also shows the proportion of Canadian workers according to the prevalence of exposure to the different dimensions of precarity. Results show, for each dimension of precarity, a graded relationship among Canadian occupations. Mean LLM exposure scores were higher for occupations with lower prevalence of exposure to precarity. The graded relationship was also reflected when examining the unadjusted mean occupational LLM exposure scores among occupations with low, medium, high and very high prevalence of precarious occupations captured in the multidimensional precarity index.

**Table 1:** Unadjusted means of occupational large language model (LLM) exposure and proportion of Canada’s labour force according to the prevalence of exposure to precarity based on data in Canada’s Labour Force Survey.

		Occupational LLM exposure value			Pro- portion
		Mean	Range		%
Temporary employment					
	1^st^ quartile (lowest exposure)	0.386	0.363–0.449		21
	2^nd^ quartile	0.378	0.305–0.424		21
	3^rd^ quartile	0.320	0.270–0.424		33
	4^th^ quartile (highest exposure)	0.275	0.209–0.317		25
Low wage					
	1^st^ quartile (lowest exposure)	0.406	0.363–0.449		21
	2^nd^ quartile	0.364	0.305–0.424		22
	3^rd^ quartile	0.347	0.27–0.424		24
	4^th^ quartile (highest exposure)	0.263	0.209–0.317		32
Irregular work hours					
	1^st^ quartile (lowest exposure)	0.504	0.473–0.535		26
	2^nd^ quartile	0.385	0.321–0.449		23
	3^rd^ quartile	0.264	0.214–0.314		22
	4^th^ quartile (highest exposure)	0.200	0.144–0.257		29
Involuntary part–time					
	1^st^ & 2^nd^ quartile (lowest exposure)	0.376	0.335–0.418		32
	3^rd^ quartile	0.364	0.307–0.421		31
	4^th^ quartile (highest exposure)	0.274	0.221–0.327		37
Multidimensional precarity index					
	Low (0 items assigned to 4^th^ quartile)	0.420	0.381–0.458		45
	Medium (1 items assigned to 4^th^ quartile)	0.283	0.236–0.330		17
	High (2 items assigned to 4^th^ quartile)	0.280	0.190–0.370		17
	Very high (3 items assigned to 4^th^ quartile)	0.240	0.174–0.305		21

**Figure 1:**
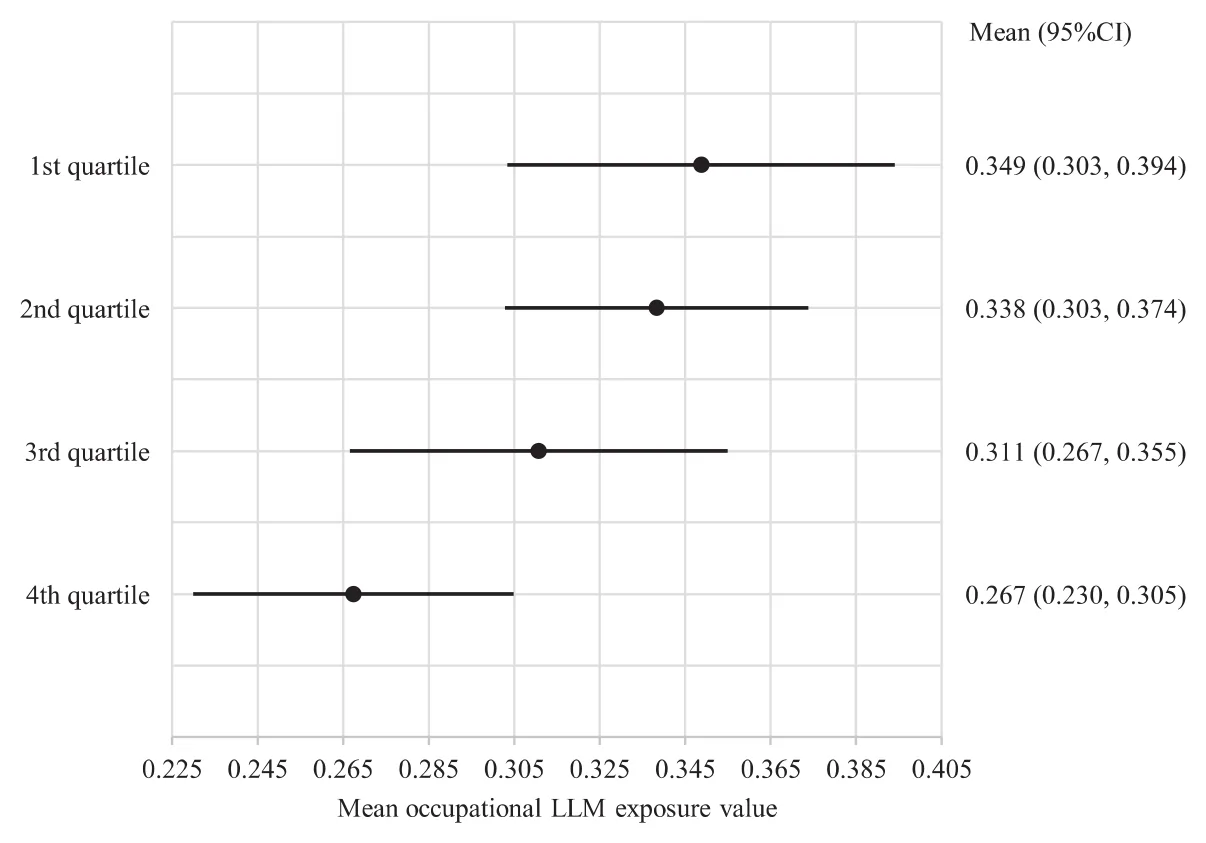
Mean occupational large language model (LLM) exposure value according to the prevalence of temporary employment using multivariable linear regression model with cluster-robust standard errors. Note: Multivariable models adjusted for gender, educational attainment, age, provincial and industry composition at the occupational level. [CI=confidence interval.]

**Figure 2:**
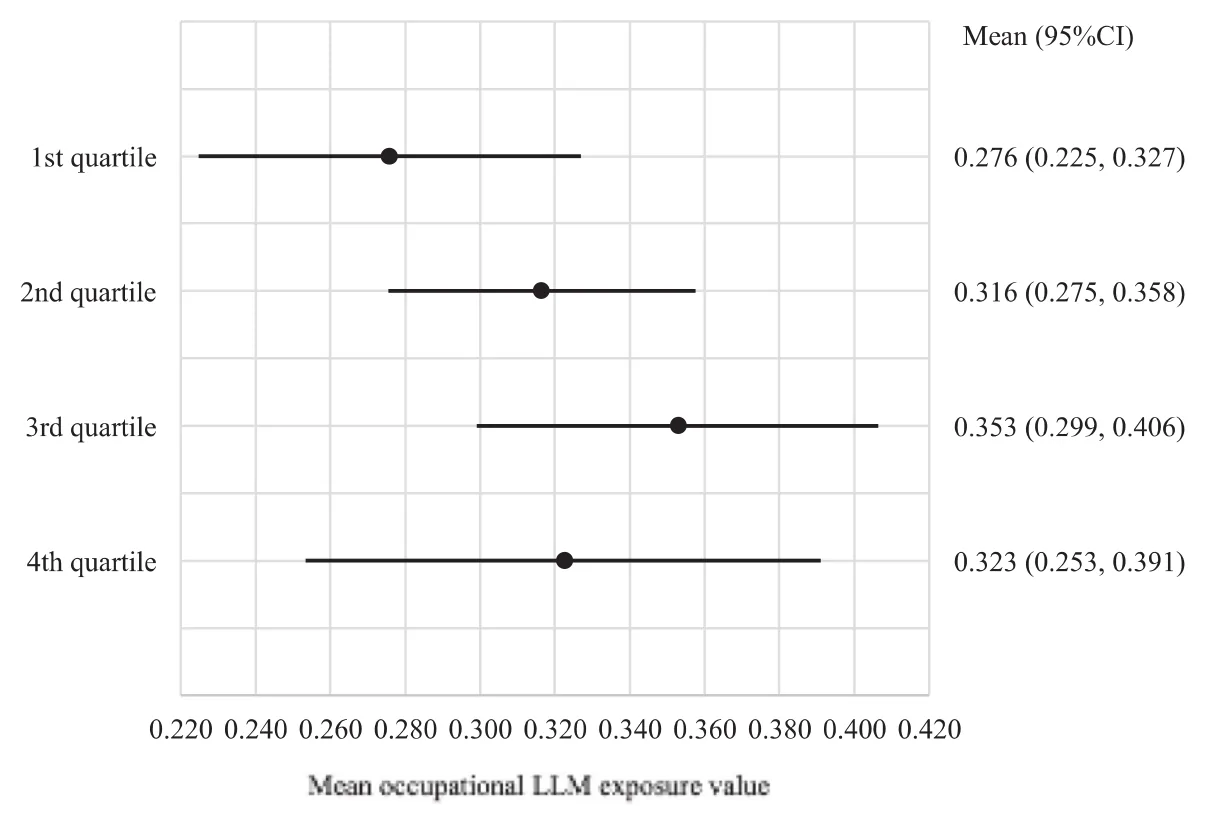
Mean occupational language model (LLM) exposure value according to the prevalence of low wage using multivariable linear regression model with cluster-robust standard errors. Note: Multivariable models adjusted for gender, educational attainment, age, provincial and industry composition at the occupational level. [CI=confidence interval.]

**Figure 3:**
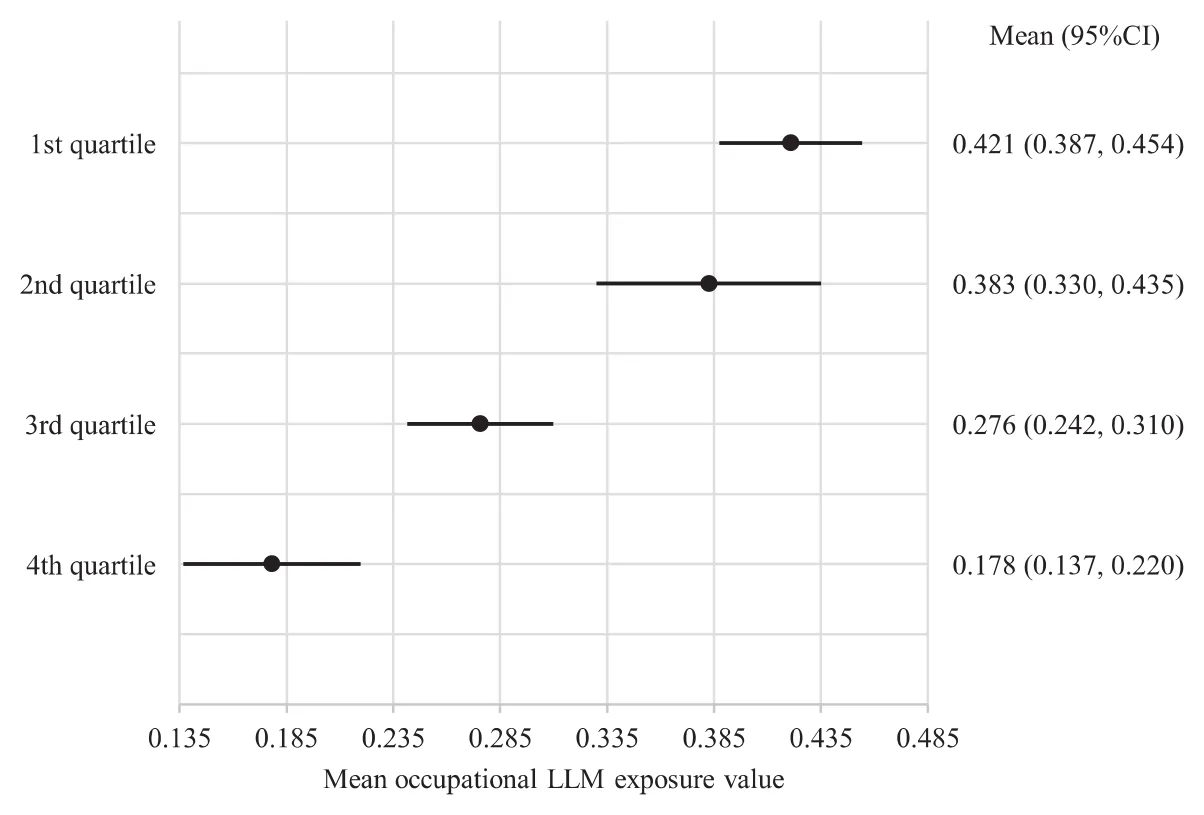
Mean occupational language model (LLM) exposure value according to the prevalence of irregular work hours using multivariable linear regression model with cluster-robust standard errors. Note: Multivariable models adjusted for gender, educational attainment, age, provincial and industry composition at the occupational level. [CI=confidence interval.]

**Figure 4:**
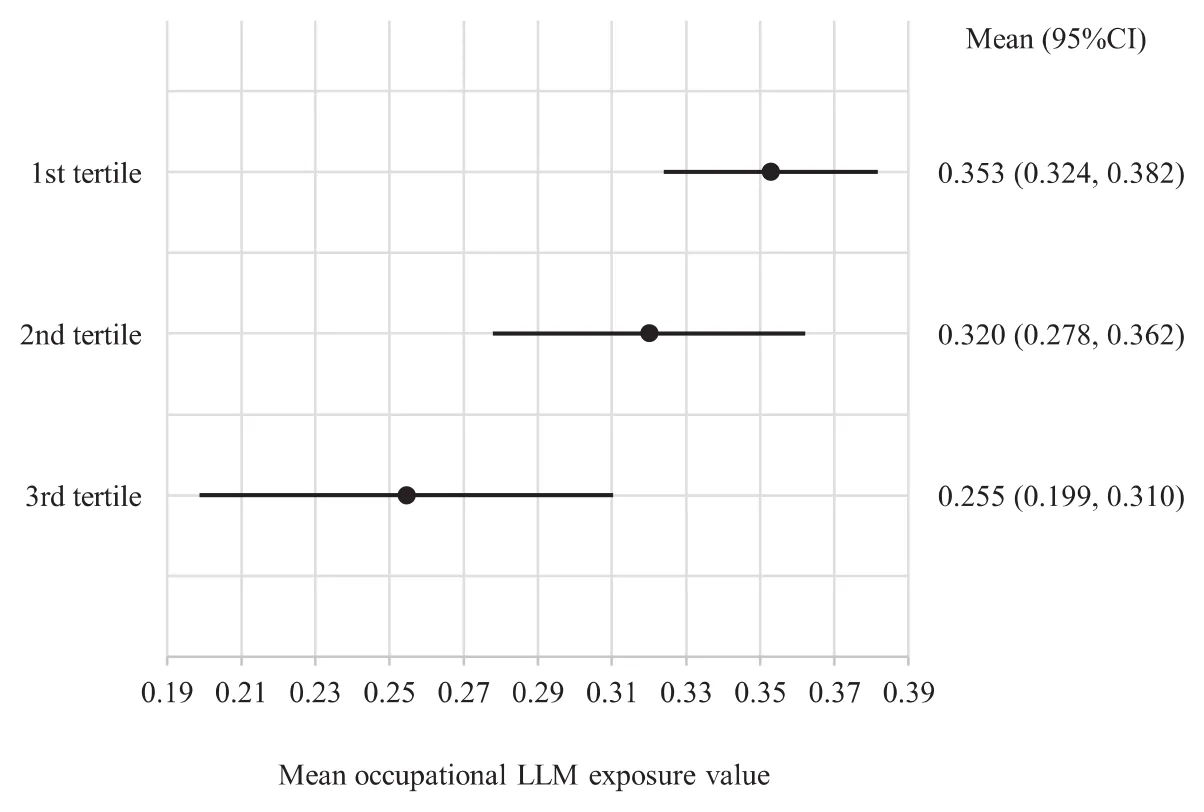
Mean occupational language model (LLM) exposure value according to the prevalence of involuntary part-time workusing multivariable linear regression model with cluster-robust standard errors. Note: Multivariable models adjusted for gender, educational attainment, age, provincial and industry composition at the occupational level. [CI=confidence interval.]

Mean LLM scores with 95% CI were estimated for each precarious occupation dimension from the multivariate linear regression models adjusting for occupational gender composition, educational attainment, age, province and industry compositions (see table 2 and figures 1–4). Additional descriptive information regarding covariates and their relationship with occupational LLM exposure are available in supplement 2. The graded relationship between prevalence of precarious occupations and LLM exposure was less prominent when adjusting for covariates in the multivariable models. When examining temporary employment (figure 1), irregular work hours (figure 3) and involuntary work hours (figure 4) our findings showed that adjusted mean LLM exposure values were significantly lower among occupations with the highest prevalence of precarity (Q1) when compared to occupations with the lowest prevalence of precarity (Q4). Adjusted mean occupational LLM exposure values did not differ significantly between the highest and lowest prevalence of low-wage work (figure 2).

**Table 2:** Univariate and multivariate linear regression models with cluster-robust standard errors examining the relationship between prevalence of exposure to precarious occupations (independent variable) and occupational LLM exposure (continuous dependent variable). [SE=standard error]

Parameter		Univariate				Multivariable ^a^		
		Estimate	SE	P-value		Estimate	SE	P-value
Model 1: Temporary employment								
	Intercept	0.386	0.030	<0.001		0.240	0.130	0.058
	2^nd^ quartile	-0.009	0.040	0.840		-0.010	0.030	0.712
	3^rd^ quartile	-0.066	0.040	0.11		-0.040	0.030	0.259
	4^th^ quartile	-0.111	0.040	<0.01		-0.080	0.030	<0.05
Model 2: Low wage								
	Intercept	0.406	0.020	<0.001		0.180	0.160	0.261
	2^nd^ quartile	-0.042	0.040	0.260		0.040	0.030	0.122
	3^rd^ quartile	-0.059	0.050	0.190		0.080	0.040	0.073
	4^th^ quartile	-0.143	0.040	<0.001		0.059	0.050	0.395
Model 3: Irregular work hours								
	Intercept	0.504	0.020	<0.001		0.330	0.130	<0.01
	2^nd^ quartile	-0.119	0.040	<0.001		-0.040	0.030	0.208
	3^rd^ quartile	-0.240	0.030	<0.001		-0.150	0.030	<0.001
	4^th^ quartile	-0.303	0.030	<0.001		-0.240	0.030	<0.001
Model 4: Involuntary part-time work								
	Intercept	0.376	0.020	<0.001		0.230	0.130	0.083
	3^rd^ quartile	-0.012	0.040	0.740		-0.030	0.030	0.237
	4^th^ quartile	-0.102	0.030	<0.01		-0.100	0.040	<0. 01
Model 5: Multidimensional precarity index								
	Intercept	0.420	0.020	<0.001		0.320	0.140	<0.05
	Medium (1 item assigned to 4^th^ quartile)	-0.136	0.030	<0.001		-0.130	0.030	<0.001
	High (2 items assigned to 4^th^ quartile)	-0.140	0.050	<0.010		-0.130	0.040	<0.01
	Very high (3 items assigned to 4^th^ quartile)	-0.180	0.040	<0.001		-0.180	0.040	<0.001

^a^ Multivariable models adjusted for gender, educational attainment, age, provincial and industry composition at the occupational level.

**Figure 5:**
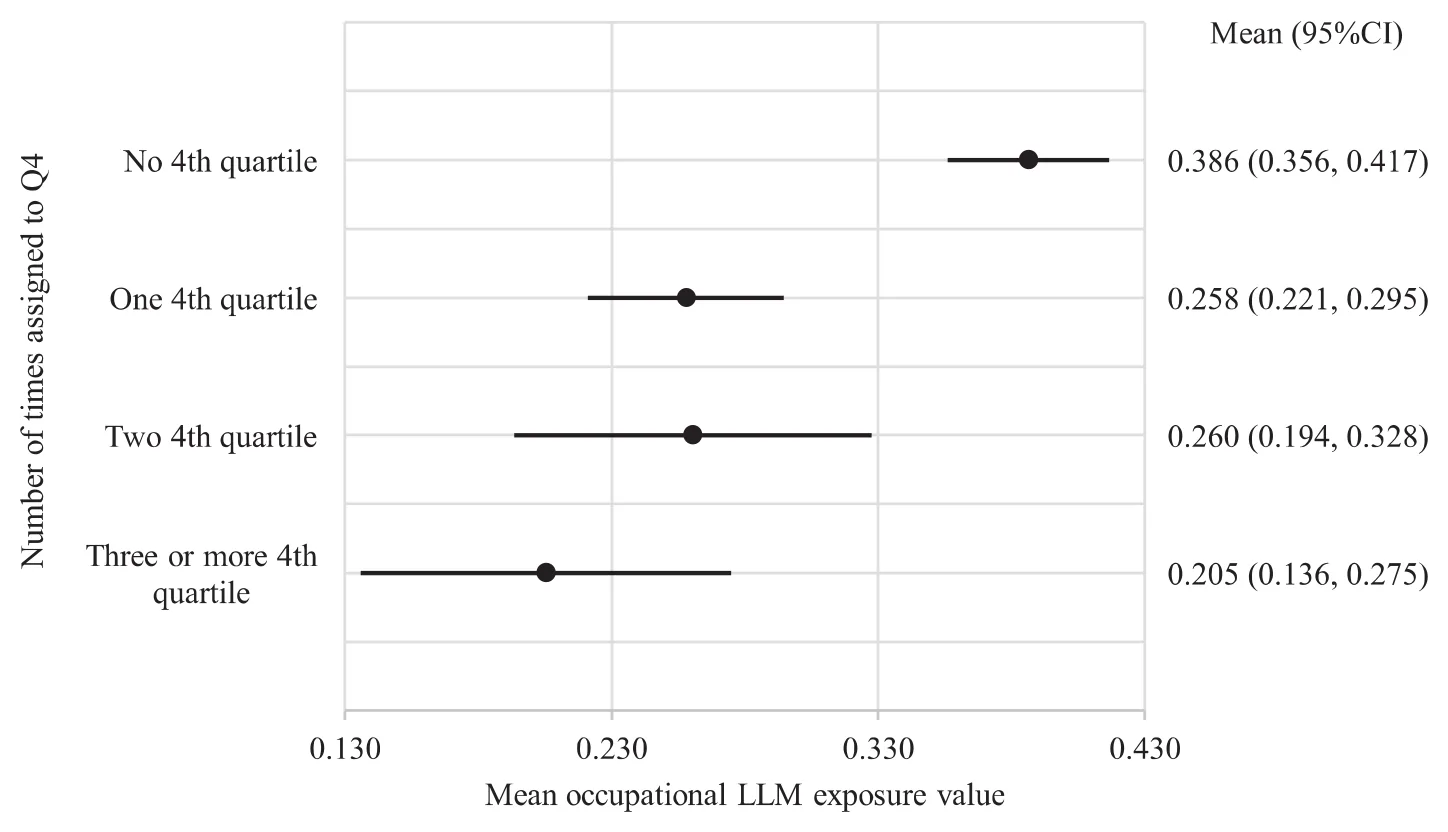
Mean occupational language model (LLM) exposure value according to the prevalence of multidimesional precarity index using multivariable linear regression model with cluster-robust standard errors. Note: Multivariable models adjusted for gender, educational attainment, age, provincial and industry composition at the occupational level. [CI=confidence interval.]

Our final multivariate linear regression model examined the relationship between the multidimensional precarity index and occupational LLM exposure (see figure 5). We found that occupations characterized by low exposure to precarity had a significantly higher adjusted mean LLM exposure value (mean 0.386, 95% CI 0.0.356–0.417) when compared to occupations characterized by medium (mean 0.258, 95% CI 0.221–0.295), high (mean 0.260, 95% CI 0.194–0.328) or very high (mean 0.205, 95% CI 0.136–0.275) precarity where there was no significantly different variation in mean estimates. Sensitivity analysis exhibited the same gradient pattern, supporting the robustness of our quartile-based analysis (see supplement 3).

## Discussion

A great deal of attention has been directed towards examining and addressing precarious occupations because of their established relationship with adverse health and economic outcomes in the working population (33, 34). To date our understanding of how the adoption of AI systems can impact different occupations including those that are characterized by precarity (35). Using a representative survey of Canadian workers, we examined four dimensions of precarious occupations and their relationship to LLM exposure. When taken together, findings indicated that the most precarious occupations were less likely to be exposed to LLM. The most precarious occupations may be those that are less likely to involve complex cognitive and analytic tasks that are codifiable and well-suited to being performed by LLM (36). When compared to past periods of technological change, we show that LLM impact a different portfolio of occupations which are potentially associated with higher job quality and may contribute to emerging health and socioeconomic implications. There is a need to build on these findings and examine the experiences and health impacts of workers exposed to LLM in their working lives.

Research conducted during past periods of technological change have indicated that the adoption of new technologies has been associated with increases in precariousness reported by workers in occupations facing the lowest quality employment conditions (5, 6). Using population-level data, our study offers a different perspective showing that when using a multidimensional measure, the most precarious Canadian occupations may be least affected by LLM. When examining specific dimensions of precarious occupations, we found that the prevalence of low wage work was associated with lower occupational LLM exposure in our univariate models but not at the multivariable level. Our univariate findings align with past studies showing a relationship between higher wages and greater occupational exposure to LLM (18, 22). Findings from the multivariate model highlight that when other occupational characteristics are adjusted for, individual dimensions of precarity may have a different relationship with LLM exposure.

The occupational LLM exposure measure and analytical approach we utilized cannot tell us how workers within each occupation are affected by the technology. Drawing from existing research and literature, findings could be interpreted in several ways. Unlike previous waves of technological change which put pressure on workers in lower skilled occupations, the use of LLM within the workplace are affecting occupations with higher skill requirements whose job tasks maybe codifiable. The result may be an increased competition among workers in high quality (least precarious) occupations and, in the long-term, may contribute to skill obsolescence, dehumanization of work, decreased wages, job insecurity and polarization in the labor market (36, 37). As AI systems like LLM become more advanced in their predictive capabilities, there could be a net increase in the number of workers in precarious occupations in the labor market who may be exposed to conditions that impact health and safety. Through this interpretation of our findings, there is a need to consider worker employment and health protections that are relevant to those in precarious occupations and those in higher quality occupations which have traditionally been sheltered from technological change.

Alternatively, those most exposed to LLM could see productivity benefits. There is evidence that the use of LLM to perform job tasks can free up time for higher value activities (38–41). These studies suggest that workers in highly skilled professional roles, such as financial analysts or healthcare providers, can use AI to handle data processing or diagnostic support, allowing them to focus on strategic thinking, decision-making, and interpersonal communication (39). At the same time, those working in precarious occupations could be excluded from the productivity benefits associated with LLM that could result in missing out on potential financial gains that can accompany the use of AI at work. Over time, the uneven exposure of occupations have to LLM and other emerging AI systems may contribute to labor market inequities and health disparities (18, 42).

To further interpret our results of aggregate and specific dimensions of precarious occupations, research using primary collected data within workplaces is required to examine how LLM impact conditions within both precarious and higher quality occupations to illustrate the benefits and harms the technology may present and subsequent implications for worker health and wellbeing. In addition, there is evidence indicating that a worker’s social position (eg, age, gender) may be associated with employment in occupations with varying levels of AI exposure and precarity (43). For instance, some early-stage research shows that younger workers, who are most likely to work precariously, are more likely to start their careers in occupations that are highly exposed generative AI which could contribute to declining rates of employment in this age group (35, 44). Primary data may help to explain how precarious occupations and their exposure to LLM can differ for specific segments of the labor market and unpack advantage or disadvantage and where additional research and policy attention can be directed.

Study strengths include the use of a representative national labor force survey to describe occupational LLM exposure and multiple precarious occupation dimensions enabled us to develop population-level estimates. Although we were not able to directly assess the impacts on health outcomes in the data, we advance research by showing the extent to which LLM at work are associated with high and low levels of precarity (4, 45). There are several study limitations to acknowledge. In the absence of direct measures, we utilized a novel measure which offered a proxy regarding the extent to which an occupation is composed of job tasks that can be performed by an LLM (18). The measure is unable to determine the realized exposure of an occupation to LLM or recent advancements in the technology. Additionally, the LLM exposure measure focused specifically on software (eg, LLM -powered chatbot or application programming interfaces) and does not examine other technologies such as robotics where there is uncertainty around reliability and adoption and where there may be difficulty in creating a measurement rubric. Moreover, findings may not account for the broader range or AI systems that can exist within different industries. Our measurement approach also does not capture how LLM impact occupations including the creation of new tasks that may emerge because of technology, productivity benefits or risks or changes to employment outcomes. Examining LLM exposure at the occupational level may not account for potential heterogeneity in an exposure such as differences in specific job tasks within occupations. Accordingly, our analytical approach may introduce ecological bias if inferences about individuals are drawn from findings presented at the occupational level. Utilizing the LFS meant that we were limited in the availability precarious occupation dimensions we examined. The addition of other dimensions of employment and working conditions could offer a more robust picture of the occupations that may be more or less precarious. Lastly, our findings provide a snapshot regarding the impact of LLM on occupations at one point in time. Innovations in LLM systems and other forms of AI may result in future innovations which may have varying impacts on different occupations.

### Concluding remarks

Working in a precarious occupation can be directly and indirectly associated with the health and safety of workers. Within the context of the growing utilization of AI within workplaces, our study leverages national population-level data of Canada’s labor market to examine the interrelationship between precarious occupations and their exposure to LLM. We show that occupations which are of the highest quality and where precariousness is lowest are also more likely to be exposed to LLM. In contrast to past periods of change, we show that a different portfolio of occupations may be most affected LLM, creating novel challenges and opportunities for different groups of workers related to the extent to which they are employed in precarious occupations. We underscore the need for additional research to unpack the impact of LLM on workers to build an evidence base that can be used to promote high quality employment and to ensure that all workers are able to capitalize on the productivity benefits of LLM while also maintaining healthy and safe working lives.

## Supplementary material

Supplementary material 1

Supplementary material 2

Supplementary material 3
