# Supplementary material for: Large language model exposure and precarious occupations: Unpacking relationships in the Canadian labor force
Source: Scand J Work Environ Health. 2026 Aug 31;52(5):615–24. doi: 10.5271/sjweh.4312 (PMC13555092; doi:10.5271/sjweh.4312)
Supplement: Supplementary file 1 [file SJWEH-52-615-S003.pdf]

Supplement 3: Sensitivity analysis of mean occupational large language model (LLM) exposure value according to the prevalence of the multidimensional precarity index using multivariable linear regression model with cluster-robust standard errors. Each precarious occupation variable was reclassified using tertiles.

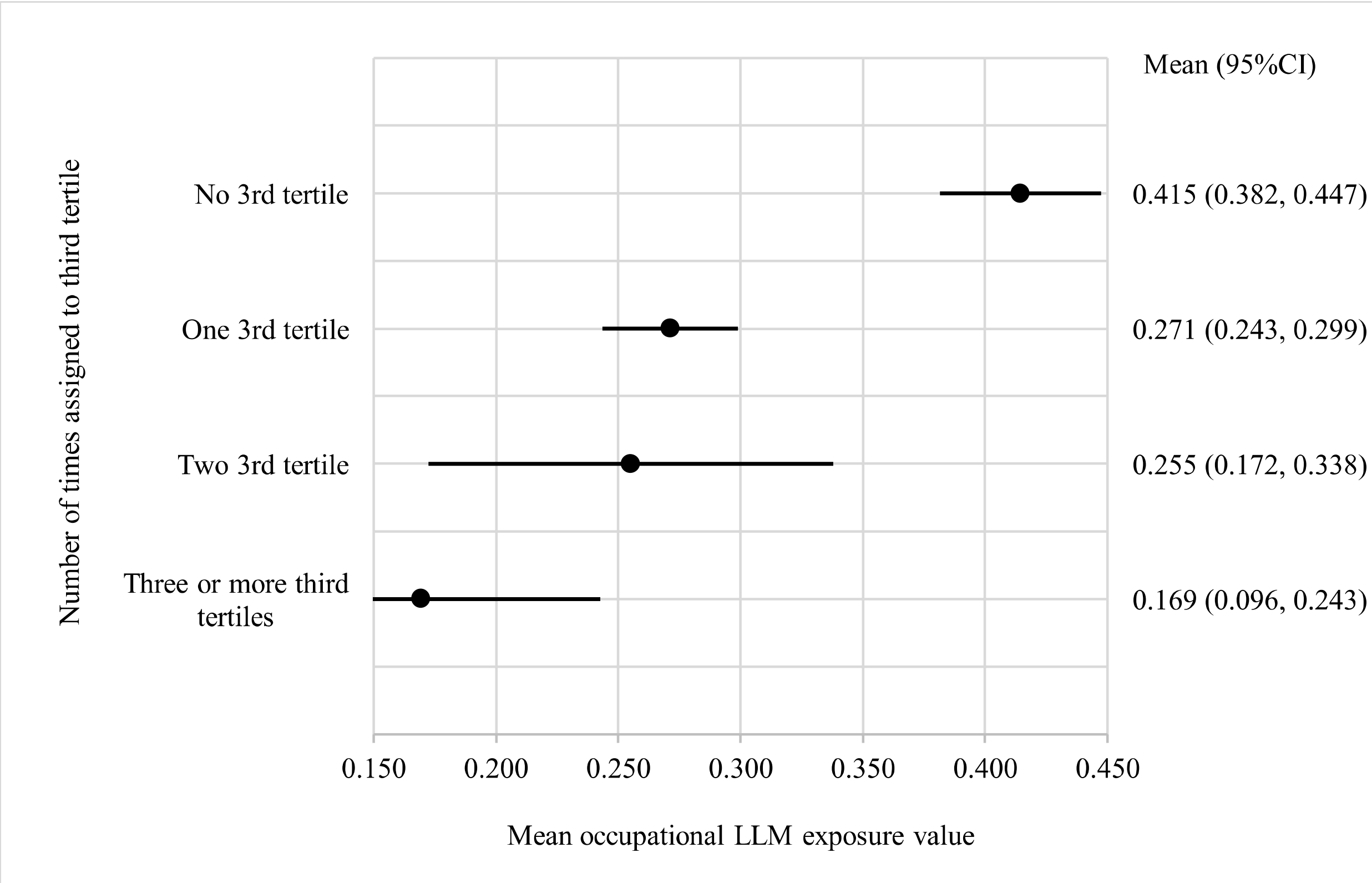


Note: Multivariable models adjusted for gender, educational attainment, age, provincial and industry composition at the occupational level; CI = confidence interval
