# Supplementary material for: Large language model exposure and precarious occupations: Unpacking relationships in the Canadian labor force
Source: Scand J Work Environ Health. 2026 Aug 31;52(5):615–24. doi: 10.5271/sjweh.4312 (PMC13555092; doi:10.5271/sjweh.4312)
Supplement: Supplementary file 2 [file SJWEH-52-615-S002.docx]

Supplement 2. Study covariates measured at the Canadian occupational level and their correlation with occupational large language model (LLM) exposure

|  |  | Correlation with occupational LLM exposure | |
| --- | --- | --- | --- |
|  | % | r | p |
| Occupational gender composition |  |  |  |
| Women | 49% | 0.289 | <0.001 |
| Occupational educational attainment composition |  |  |  |
| <Some post-secondary education | 30% | -0.504 | <0.001 |
| Trades certification or diploma | 9% | -0.512 | <0.001 |
| College or bachelor’s degree | 49% | 0.533 | <0.001 |
| University degree above bachelor’s degree | 11% | 0.453 | <0.001 |
| Occupational age (years) breakdown |  |  |  |
| 15-24 | 15% | -0.312 | <0.001 |
| 25-34 | 24% | 0.236 | <0.001 |
| 35-44 | 22% | 0.389 | <0.001 |
| 45-54 | 20% | 0.198 | <0.001 |
| 55+ | 19% | -0.115 | <0.010 |
| Provincial composition |  |  |  |
| Ontario | 1% | -0.182 | <0.001 |
| Newfoundland | 0.4% | -0.141 | <0.01 |
| Prince Edward Island | 3% | -0.155 | <0.001 |
| Nova Scotia | 2% | -0.180 | <0.001 |
| New Brunswick | 23% | -0.028 | <0.001 |
| Quebec | 39% | 0.316 | <0.001 |
| Manitoba | 4% | -0.349 | <0.001 |
| Saskatchewan | 3% | -0.311 | <0.001 |
| Alberta | 12% | -0.163 | <0.001 |
| British Columbia | 14% | 0.017 | 0.698 |
| Industry group composition^†^ |  |  |  |
| Goods producing industries | 21% | - |  |
| Service producing industries | 79% | 0.365 | <0.001 |
| Note: † = industry categorization using the North American Industry Classification System (NAICS) (goods-producing [11-33] or service-producing industry [41-91]) | | | |
